# Adenosine Actions on Oligodendroglia and Myelination in Autism Spectrum Disorder

**DOI:** 10.3389/fncel.2018.00482

**Published:** 2018-12-07

**Authors:** Hai-Ying Shen, Nanxin Huang, Jesica Reemmer, Lan Xiao

**Affiliations:** ^1^Robert Stone Dow Neurobiology Department, Legacy Research Institute, Legacy Health, Portland, OR, United States; ^2^Department of Integrative Physiology and Neuroscience, Washington State University, Pullman, WA, United States; ^3^Department of Histology and Embryology, Chongqing Key Laboratory of Neurobiology, Army Medical University (Third Military Medical University), Chongqing, China

**Keywords:** adenosine receptor, oligodendroglial differentiation, demyelination, neurotransmitter, autism

## Abstract

Autism spectrum disorder (ASD) is the most commonly diagnosed neurodevelopmental disorder. Independent of neuronal dysfunction, ASD and its associated comorbidities have been linked to hypomyelination and oligodendroglial dysfunction. Additionally, the neuromodulator adenosine has been shown to affect certain ASD comorbidities and symptoms, such as epilepsy, impairment of cognitive function, and anxiety. Adenosine is both directly and indirectly responsible for regulating the development of oligodendroglia and myelination through its interaction with, and modulation of, several neurotransmitters, including glutamate, dopamine, and serotonin. In this review, we will focus on the recent discoveries in adenosine interaction with physiological and pathophysiological activities of oligodendroglia and myelination, as well as ASD-related aspects of adenosine actions on neuroprotection and neuroinflammation. Moreover, we will discuss the potential therapeutic value and clinical approaches of adenosine manipulation against hypomyelination in ASD.

## Introduction

Autism spectrum disorder is a range of neurodevelopmental conditions characterized by impairments in verbal and non-verbal communications, social skills, and repetitive behaviors. The variety of presentations of ASD is due to complex combinations of environmental and genetic influences; indeed, the term spectrum itself reveals the diversity of strengths and differences in symptoms of each autism patient. According to the reports, the average prevalence of individuals suffering from ASD is about 1% of people worldwide (Murray et al., 2014). ASD usually begins in childhood and tends to persist into adulthood, sometimes causing severe disabilities that call for life-long care and support.

Although the underlying mechanisms of ASD remain unclear, the most common hypothesis has been that of disrupted cerebral connectivity during brain development – this model proposes that ASD patients have reduced connections between distant brain regions and increased connections within local regions; such a disarranged connectivity could underlie the observed abnormalities in social, cognitive, and behavioral functions (Belmonte et al., 2004). During early brain development, aberrations appear in cytoarchitectural organizations in the frontal lobe, parieto-temporal lobe, subcortical limbic structures and cerebellum (Fombonne et al., 1999; Bolton et al., 2001; Courchesne, 2004; Hazlett et al., 2005). In addition, it has further been shown that ASD patients experience multiregional abnormalities in neurogenesis, neuronal migration, and maturation (Wegiel et al., 2010; Chen et al., 2015). However, the neuronal connectivity hypothesis of ASD is not fully supported, due to inconsistent data that reveals long-range hyper-connectivity or mixed patterns of both hypo- and hyper-connectivity (Mizuno et al., 2006; Turner et al., 2006; Noonan et al., 2009; Shih et al., 2010). Recently, increasing evidence suggests that white matter and myelin abnormalities are more relevantly involved in ASD pathophysiology. For example, neuroimaging studies utilizing diffusion tensor imaging (DTI) and magnetic resonance imaging (MRI) show that white matter disruption occurs in brain regions of children with ASD (Carmody and Lewis, 2010). Molecular genetic studies also reveal aberrations of myelin-related genes in ASD patients, both in expression level and epigenetic regulation (Richetto et al., 2017). Moreover, chromatin-remodeling protein CHD8, an ASD susceptibility gene, has also been found to play a vital role in oligodendroglial development and remyelination (Zhao et al., 2018).

In addition to studies focusing directly on structural variation, there coexists exciting research into the purine ribonucleoside adenosine; as a neuro- and glia-modulator, adenosine can directly and indirectly interact with several neurotransmitters, including glutamate, dopamine, and GABA, to regulate the development of oligodendroglial cells and myelination (Stevens et al., 2002). Also, by modulating neurotransmitters, adenosine has been shown to affect certain ASD comorbidities and symptoms, such as epilepsy, impaired cognitive functions, and anxiety (Fredholm et al., 2005a,b). In this review, we will summarize and discuss the interaction between adenosine signaling system in the central nervous system (CNS), mainly focusing on its effect in oligodendrocytes and the clinical symptoms of ASD, as well as the related cellular and molecular abnormalities. We suggest that augmentation of the useful actions of adenosine may become a potential therapeutic approach for the treatment of ASD by affecting myelination.

## Adenosine Metabolism and Adenosine Receptors

Adenosine is ubiquitously found in CNS. Early studies implied that adenosine was merely a metabolite of ATP and cAMP; now adenosine is widely recognized as a neuromodulator, gliomodulator, and modulator of complex behaviors with a broad range of biological and pathological functions. Indeed, adenosine plays two important roles in the CNS: *(i)* as a homeostatic transcellular messenger between neuronal and glial cells and *(ii)* as a modulator controlling neurotransmitter release and reuptake for neuronal excitability (Shen and Chen, 2009; Boison and Shen, 2010; Shen et al., 2012).

### Adenosine Metabolism

**FIGURE 1:**
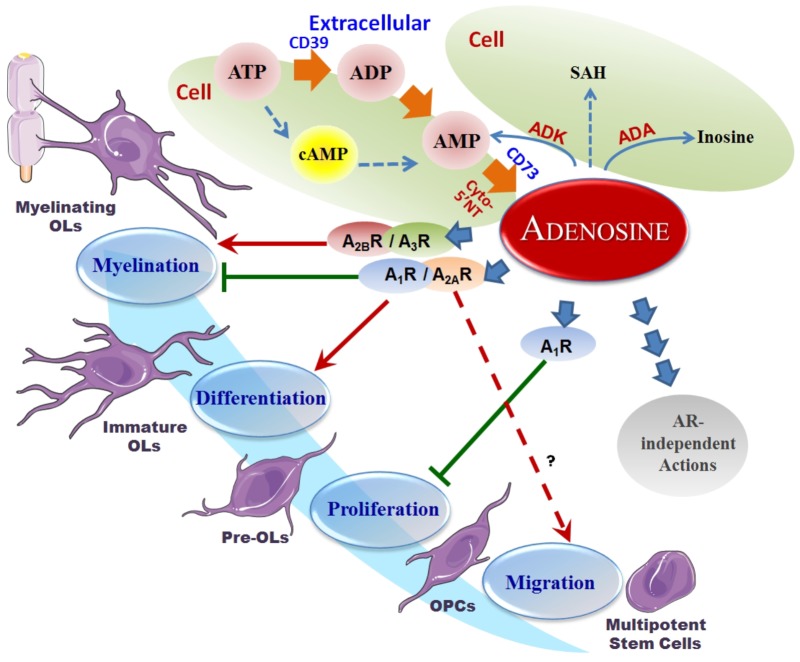
Adenosine metabolism and its effects on OPCs. Overview of the adenosine metabolism and adenosine receptor-induced actions on oligodendroglial development. It is seen that A_1_R inhibits oligodendroglial proliferation, A_1_R and A_2A_R promote differentiation and inhibit myelination, and A_2B_R and A_3_R promote myelination in the lifecycle of oligodendrocytes. The pool of extracellular adenosine is responsible for the activation of these various ARs, while itself being the subject of supply by the ATP cycle, metabolism by hydrolysis or deamination, as well as transport and internalization. Red arrows represent a stimulation effect; Green bars represent an inhibition effect; Blue arrows show the directionality of effects.

Adenosine metabolism in the CNS has been extensively reviewed elsewhere (Cunha, 2001; Latini and Pedata, 2001; Fredholm et al., 2005a,b). Unlike classical neurotransmitters, which are stored in synaptic vesicles and released by exocytosis for exclusive actions on synapses (Fredholm et al., 2005a,b), adenosine distributes extra- and intra-cellularly throughout tissues in the CNS and maintains a basal level in the range of 50–200 nM (Latini and Pedata, 2001). The intra- and extra-cellular adenosine levels exist in a state of dynamic exchange due to the influence of both equilibrative and concentrative nucleoside transporters (Baldwin et al., 2004; Gray et al., 2004). Additionally, metabolic enzymes such as ADA, ADK, SAH-hydrolase, and NTs, continually cycle adenosine through a variety of pathways (Boison and Shen, 2010). The input sources of adenosine are also varied, though strictly dependent on the metabolic state of the cell. Extracellularly, adenosine is produced by the breakdown of adenine nucleotides (such as ATP) by a variety of ecto-NTs, which include ecto-nucleoside triphosphate diphosphohydrolase CD39 and the 5′-nucleotidase CD73. Intracellularly, adenosine can be generated via the dephosphorylation of AMP or the hydrolysis of SAH by cytosolic enzymes 5′-NT or SAH-hydrolase, respectively (Schubert et al., 1979; Broch and Ueland, 1980) (Figure 1).

In the CNS, adenosine levels are largely regulated by glial cells, and two main types of equilibrative adenosine transporters that exist in astrocytic membranes. Intracellular removal of adenosine is executed by degradation to inosine or phosphorylation to AMP by ADA and ADK, respectively (Pak et al., 1994; Boison et al., 2010). The release and uptake of adenosine are mediated by bi-directional nucleoside transporters, depending on the adenosine concentration gradient between the extracellular and intracellular spaces (Gu et al., 1995). It is also worth pointing out that of adenosine levels between extracellular and intracellular spaces are usually maintained in the same range due to highly efficient cellular equilibrative nucleoside transporters in most cells for facilitated diffusion (Cass et al., 1999).

### Adenosine Receptors and Neurotransmissions

As a key neurotransmitter, adenosine signaling plays a significant role in neuronal excitability and neuroprotection, as well as synaptic and non-synaptic neurotransmission in the CNS. The adenosinergic system also interacts with several other major neurotransmitters, such as dopamine, glutamate, acetylcholine noradrenaline, serotonin (5-hydroxytryptamine), and endocannabinoids (Sperlagh and Vizi, 2011; Shen et al., 2013); these interactions have, however, been reviewed and discussed extensively (Sebastiao and Ribeiro, 2000; Fredholm et al., 2005a,b; Chen et al., 2013). Of particular interest in this review is the ability of adenosine to modulate neurotransmitter release and neuronal excitability in the CNS via activation of four different subtypes of G-protein coupled adenosine receptors (ARs): A_1_R, A_2A_R, A_2B_R, and A_3_R (Fredholm et al., 2005a,b). Through ARs, adenosine influences a wide range of brain functions under physiological and pathophysiological conditions, including neuroprotection, neuroinflammation, sleep, psycholocomotion, anxiety, cognition and memory, and neuropsychological disorders (Dunwiddie and Masino, 2001; Fredholm et al., 2005a,b; Shen et al., 2008, 2012). The interactions among subtypes of ARs and between adenosinergic signaling and other neurotransmissions may involve molecular mechanisms at multiple levels, such as *(i)* direct cross-talk between receptors on the cell membrane, *(ii)* downstream intracellular second messenger systems among different receptors, *(iii)* trans-synaptic actions, and *(iv)* interactions between neurons and glial cells (Sebastiao and Ribeiro, 2000; Stevens et al., 2002; Dare et al., 2007; Shen and Chen, 2009; Boison et al., 2010).

Through AR-dependent actions, adenosine modulates the release of several neurotransmitters in the CNS, including dopamine, glutamate, GABA, serotonin, noradrenaline, acetylcholine, and cannabinoids (Fredholm and Dunwiddie, 1988; Cunha, 2001; Dunwiddie and Masino, 2001). Among the four subtypes of ARs, A_2A_R and A_1_R play the most significant roles in the interaction between the adenosinergic signaling pathway and other neurotransmitters, while A_2A_R and A_3_R have a limited effect (Fredholm et al., 2005a,b; Chen et al., 2013). Even so, the AR-mediated manipulations on neurotransmissions involve a high degree of complexity (Dixon et al., 1997; O’Kane and Stone, 1998; Lopes et al., 1999; Gorter et al., 2002). Firstly, this regulation is primarily mediated by the interplay of A_1_R and A_2A_Rs, as these two subtypes have different partnering sets of G-proteins mediating opposing excitatory vs. inhibitory actions. Specifically, the activation of G_i/o_-coupled A_1_R (and A_3_R) mediates the adenosine-dependent inhibition of neurotransmitter release at pre-synaptic neuronal terminals and represses post-synaptic neuronal (Snyder, 1985; Fredholm and Dunwiddie, 1988; Latini et al., 1996). Contrastingly, activation of G_s/olf_-coupled A_2A_R (and A_2B_R) exerts excitatory activity of stimulating the release of glutamate, acetylcholine, and GABA in the striatum and hippocampus (Kirkpatrick and Richardson, 1993; Kirk and Richardson, 1994; Cunha et al., 1995a,b; Sebastiao and Ribeiro, 1996). Secondly, the actions from pre- and post-synaptic sites also contribute to the complexity of AR actions. Presynaptically, stimulation of A_1_R suppresses the release of transmitters including dopamine, glutamate, 5-HT, acetylcholine, GABA, and noradrenaline; whereas activation of presynaptic A_2A_R facilitates the release of acetylcholine, GABA, and glutamate (Sperlagh and Vizi, 2011). Post-synaptically, activation of A_2A_R inhibits activity of dopamine D_2_ receptor (D_2_ R), while A_1_R activation counteracts functions of dopamine D_1_ receptor (D_1_R) (Ferre et al., 1991; Fredholm et al., 2005a). A_2A_Rs serve a permissive role in activating cannabinoid receptor (type 1) (CB1R)-mediated inhibition of excitatory transmission, while A_1_R prevents the CB1R-mediated reduction of glutamate release. In addition, adenosine signaling affects the activity of post-synaptic AMPAR and NMDAR by control of glutamate release (Sebastiao and Ribeiro, 2009; Sperlagh and Vizi, 2011). Thirdly, the formation of these AR heteromeric receptor complexes also allows for their regulation at the second messenger level. For example, ARs can form heteromers between ARs themselves or with other receptor types, such as glutamate receptors, dopamine D_1_ and D_2_ receptors, or cannabinoid receptors. While the A_2A_R-D_2_R heteromers are the first demonstrated epitope–epitope electrostatic interactions underlying receptor heteromerization (Ferre et al., 2004), the capability of A_1_R and A_2A_R to form homo- or hetero-oligomers with both other receptors and ARs alike has widened their biological roles in developmental, physiological, and pathological situations. In addition to AR actions, studies suggest that adenosine regulating enzymes may also affect epigenetic modifications (Kiese et al., 2016), including alteration of transmethylation and histone modifications (Borea et al., 2016). All of the above regulatory levels comprise a high degree of complexity of adenosinergic actions.

## Adenosine’s Effect on Oligodendroglial Development

In the CNS, the development of the oligodendrocytes lineages (OLs) is a crucial step for myelination, which is essential for the normal functioning of transduction between neurons. Adenosine actions play a crucial role in this process (Fields, 2006) via AR-mediated direct effects and/or actions from AR-interacted neurotransmissions. Prior to myelination, oligodendroglial cells pass through distinct stages, including OPCs, pre-mature OLs, and finally mature OLs. It has been shown that all subtypes of ARs (i.e., A_1_R, A_2A_R, A_2B_R, and A_3_R), along with ADA, ADK, and the equilibrative nucleoside transporters, ENT1 and ENT2, are expressed on oligodendroglial cells (Gonzalez-Fernandez et al., 2014). Adenosinergic signaling has various effects on oligodendroglia *via* the activation of ARs and changes in adenosine metabolism (Stevens et al., 2002), while adenosine actions on oligodendroglial development and OL maturation are AR subtype-dependent (Burnstock et al., 2011a,b). For example, adenosine can act through A_1_R activation to inhibit the proliferation of OPC but also can promote the differentiation of OPCs into myelinating OLs (Stevens et al., 2002; Asghari et al., 2013). In contrast, adenosine acting through A_2A_R activation can inhibit OL maturation, whereas inactivation of A_2A_R can delay the differentiation of OPCs *via* altered rectifier potassium currents in culture (Coppi et al., 2013). Also, the A_2A_R agonist CGS-21680 can mediate OPC differentiation, which in turn can be completely blocked by the selective A_2A_R antagonist SCH-58261. In addition, the activation of A_3_R by its agonist 2-CI-IB-MECA can cause concentration-dependent oligodendroglial cell death. This mechanism is ultimately due to disruption of mitochondrial membrane potential in oligodendroglial cells; through activation of Bax and Puma proapoptotic proteins, A_3_R mediates apoptosis and necrosis (Gonzalez-Fernandez et al., 2014). Nevertheless, adenosine is suggested to not only inhibit proliferation of OPCs, but also stimulate the migration and differentiation of OPCs and promote myelin formation (Stevens et al., 2002; Othman et al., 2003; Karadottir and Attwell, 2007); further studies are still needed to dissect these seemingly contradictory roles of ARs and adenosine modulating enzymes.

## Neurotransmitter-Mediated Actions of Adenosine on Oligodendroglial Development

### Glutamatergic Effects

The proliferation and differentiation of OPCs, as well as their capability to myelinate axons, are partly controlled by neurotransmitters linked to adenosine modulation; the activation of these neurotransmitter receptors contributes to the damage of OLs and disrupted myelination in pathological scenarios (Karadottir and Attwell, 2007; Kolodziejczyk et al., 2009; Hamilton et al., 2017). For example, glutamate receptors are expressed in oligodendroglial cells and affect their development (Karadottir et al., 2005). Under normal physiological conditions, glutamate acts through the activation of NMDAR to increase OPC migration, whereas activation of the AMPAR or kainate receptors (but not NMDAR) decreases the proliferation of OPCs (Karadottir and Attwell, 2007) by altering both intracellular sodium concentration and potassium channel function. Blocking NMDA receptors with phencyclidine will result in the inability of OLs to mature (Lindahl et al., 2008).

### Dopaminergic and Other Neurotransmitters’ Effects

Dopamine has been reported to modulate CNS myelination (Belachew et al., 1999) through underlying mechanisms that are not fully elucidated. It was found that the dopamine antagonist haloperidol increases OPC proliferation but inhibits their differentiation, an action likely to occur *via* D_3_R (Bongarzone et al., 1998). To clarify the effect of the different subtypes of dopaminergic receptors involved in oligodendroglial development, genetic approaches may need to be utilized in the future. OPCs also express acetylcholine receptors, and the activation of muscarinic acetylcholine M3 receptors increases OPC proliferation through activation of the MAPK signaling pathway; conversely, anti-muscarinic treatment has been seen to accelerate functional myelin repair (Abiraman et al., 2015). Meanwhile, inactivation of nicotinic acetylcholine receptors (nAChR) can inhibit proliferation of OPCs, as evidenced by the ability of the nAChR antagonist mecamylamine to suppress OPC differentiation (Imamura et al., 2015).

Together, while the adenosinergic system plays crucial roles in glutamatergic and dopaminergic neurotransmissions at multiple molecular and cellular levels (Stevens et al., 2002; Barnea-Goraly et al., 2004; Matos et al., 2015), the studies in this section suggest that adenosine signaling can influence the regulation of oligodendroglial development and remyelination via neurotransmitters’ actions itself.

## Adenosine and Adenosine Receptors in Demyelination and Remyelination

Various studies have shown that myelin abnormalities are involved in ASD, affecting information processing and cognition. Researchers utilized MRI scans to compare myelination between ASD and neurotypical children, and found that children with ASD had more myelinated neural fiber than the average level of similarly aged children in both the left and right medial frontal cortex, but had less myelination in the left temporoparietal junction (Deoni et al., 2015). Another study using DTI found that the fractional anisotropy value – an index used to describe the statistical difference in white matter brain scanning – was reduced in the white matter in children and adolescents with autism compared to controls, including regions adjacent to the superior temporal sulcus, ventromedial prefrontal cortices, anterior gyri, and temporoparietal junctions and corpus callosum, as well as regions adjacent to the temporal lobes close to the amygdala. This suggests that white matter disruption between brain regions may cause impaired social cognition in autism (Ginsberg et al., 2012). Measurement of myelin content by performing cross-sectional imaging of fractional anisotropy – based on the movements of water molecules – showed that the global myelin water fraction was significantly lower in autism patients than in neurotypical controls (Stolerman et al., 2016). By applying whole genome DNA methylation microarrays analysis and high-resolution whole-genome gene expression, researchers have found that changes in myelin and myelination-related genes were associated with specific behavioral domains of autism (Setzu et al., 2006). Moreover, genome-wide transcriptional profiling techniques in combination with MRI and epigenetic analyses reveal that viral-like prenatal immune activation leads to myelin-related epigenetic and transcriptional changes, which may lead to neurodevelopmental disorders like autism (Richetto et al., 2017). Recently, mutation of the chromatin remodeler protein CHD8, which is crucial for oligodendroglial development, was found to be associated with ASD (Setzu et al., 2004). Taken together, these research results suggest that white matter abnormalities may contribute to ASD pathogenesis, and targeting oligodendroglia may represent a new therapeutic strategy for ASD.

Endogenous remyelination usually occurs after demyelination, through the differentiation of OPCs and thus reproduction of myelin around the demyelinated axons (Back and Rosenberg, 2014; Safarzadeh et al., 2016). A successful remyelination is determined by the capability of OPCs to proliferate and differentiate into myelinating OLs, and these processes are also affected by the adenosine system (Fields, 2006). Accumulating evidence indicates that the adenosinergic signaling system is involved in immunity and inflammation of demyelinating diseases (Sebastiao and Ribeiro, 2009; Ginsberg et al., 2012), likely *via* ARs (A_1_R, A_2A_R, A_2B_R, and A_3_R) on the surface of immune cells (Tsutsui et al., 2004). For instance, white matter injury (WMI) in premature newborn and embryos has been found to be closely related with adenosine (Johnston et al., 2001). Another typical example of such demyelinating diseases is MS, which is an autoimmune-mediated inflammatory disease characterized by multifocal demyelination that is associated with myelin destruction, oligodendroglial cell death, and axonal degeneration.

### Adenosine A_1_ Receptor

Clinical and experimental evidence indicates that A_1_R plays a role in the modulation of neuroinflammation (Si et al., 1996). A decreased expression of A_1_R in peripheral mononuclear cells and decreased adenosine levels in plasma were seen in patients with MS. In addition, expression of A_1_Rs on CD45^+^ glial cells was decreased by 50% in MS patients and similar observations have been made in patients with other CNS diseases (Haselkorn et al., 2010). From animal studies it was shown that pharmacological activation of A_1_R by N6-cyclohexyladenosine (CHA) can induce remyelination and protect existing myelin in a rat model of optic chiasm demyelination. Further study revealed that this protective effect on myelinating cells is mediated by A_1_R by potentiating regeneration of endogenous neural progenitors (Asghari et al., 2013). Apparently, activation of A_1_Rs is an important inhibitory mechanism against neuroinflammation in MS and has also been observed to attenuate neuroinflammation and demyelination in EAEs animal models (Si et al., 1996; Yao et al., 2012). In accordance with these findings, studies have revealed that inactivation of A_1_R resulted in a progressive-relapsing form of EAE in A_1_R KO mice, with worsened demyelination and axonal injury, increased microglial proliferation, and enhanced expression of matrix metalloproteinase-12 (MMP-12), iNOS, as well as proinflammatory gene interleukin-1beta (IL-1β) (Si et al., 1996). The modulation of microglial proliferation was also seen in pharmacological approaches, as both non-selective and selective A_1_R agonists attenuated the proliferation of rat microglia, and activation of A_1_R was shown as an endogenous inhibitor of the microglial response (Turner et al., 2002; Fogal et al., 2010). Therefore, the interplay between A_1_R and neuroinflammation is a reciprocal regulatory mechanism; namely, microglial A_1_R is downregulated during neuroinflammation in animal models of EAE and patients with MS (Hasko and Pacher, 2008), and the inactivation of A_1_R, in turn, exacerbates EAE and MS progression. Thus, modulation of neuroinflammation by A_1_R manipulation may offer interesting therapeutic opportunities for MS and other demyelinating diseases (Yao et al., 2012). In developmental stages, however, adenosine acts via A_1_Rs and induced diffused WMI. Immature OLs express more A_1_Rs compared with mature cells *in vitro*. Adenosine plays an important role in OL lineage progression by causing OL precursor cells to differentiate prematurely. A_1_R stimulation induces white matter loss and mediates hypoxia-induced ventriculomegaly (Mayne et al., 2001). Aiming at modulating this process may provide a novel strategy for WMI treatment (Sitkovsky et al., 2004).

### Adenosine A_2A_ Receptor

A_2A_R also exerts significant anti-immune and anti-inflammatory actions on immune cells (Vincenzi et al., 2013; Ingwersen et al., 2016; Liu et al., 2018). In human studies, A_2A_Rs are up-regulated in MS patients and administration of the A_2A_R agonist CGS-21680 can reduce lymphocyte proliferation of MS patients. This activation of A_2A_R mediates a significant inhibition of tumor necrosis factor alpha (TNF-α), IL-6, IL-1β, IL-17, interferon gamma (IFN-γ), and represses cell proliferation, expression of very late antigen (VLA)-4, and activation of NF-kB (Genovese et al., 2009). Further research has been conducted in animal models to assess the pattern of A_2A_R expression in neuropathologically damaged tissue. A_2A_R is seen to be expressed in infiltrating immune cells, as well as the surrounding endothelium; it is also up-regulated on T cells and macrophages/microglia in EAE lesions (Mills et al., 2008). Likewise, endothelial A_2A_R is detected in demyelinated areas in MS brain samples (Mills et al., 2008). Moreover, A_2A_R agonist CGS-21680 can ameliorate EAE neurological deficiencies in mice. In sum, A_2A_R has been shown to have an overall influence in mediating BBB function in CNS demyelinating diseases (Matos et al., 2013).

Activation of A_2A_R *in vitro* inhibits the migration of CD4^+^ T cells, macrophages, and primary microglia, and suppresses macrophage/primary microglia-mediated phagocytosis of myelin. Along with this finding, A_2A_R-specific agonists have also been seen to inhibit myelin-specific T cell proliferation *ex vivo* and ameliorate EAE (Mills et al., 2008). A_2A_R agonists suppressed *in vivo* primary mechanical injury and secondary inflammatory tissue damage after spinal cord injury; they can also reduce leukocyte recruitment and the activation of the JNK signaling pathway in oligodendroglia, thus protecting the spinal cord from injury-induced demyelination (Paterniti et al., 2011). In contrast, A_2A_R KO mice develop increased morbidity, exacerbated neurobehavioral deficits, and generally more severe EAE pathology than their WT littermates. A_2A_R KO mice exhibit a severe phenotype of demyelination in EAE with enhanced infiltration of inflammatory cells in the spinal cord and cerebral cortex, accompanied by reduced expression of anti-inflammatory cytokines and increased expression of pro-inflammatory cytokines in the CNS and blood (Hasko and Pacher, 2008). However, studies also demonstrated that there is a dual role of A_2A_R in autoimmune neuroinflammation; for instance, A_2A_R-specific agonists inhibited the proliferation of myelin-specific T cell *ex vivo* and ameliorated EAE, whereas application of the same agonist after the onset of disease exacerbated progression of non-remitting EAE. This suggests that activation of A_2A_R exerts complex effects on chronic autoimmune neurodegeneration (Wei et al., 2013). The paradoxical effects of A_2A_R may be dependent on pathophysiological conditions; further studies are needed to clarify the manipulatory role of A_2A_R on demyelinating disorders as well as the potential shifting of the predominant role of A_1_R and A_2A_R in the pathogenesis of demyelination.

In developmental models, adenosine signaling pathway acts via A_2A_R to inhibit OPC differentiation (Coppi et al., 2013). Increased nitric oxide release by microglia, up-regulated neuronal glutamate release, and decreased glutamate uptake by astrocytes are all results of A_2A_R activation, and leads to aggravated excitotoxicity (Cook, 1990). Blockade of A_2A_R, on the other hand, has been revealed to have a negative effect on demyelination in the spinal cord injury model (Masino et al., 2011).

### Adenosine A_2B_ and A_3_ Receptors

The A_2B_R is a relatively low-affinity adenosine receptor compared to A_1_R and A_2A_R; in fact, the activation of A_2B_R requires a pathologically enhanced level of adenosine (Fredholm et al., 2005a; Chen et al., 2013). A clinical study revealed upregulated A_2B_R in the peripheral leukocytes of MS patients; this is in line with findings which showed increased A_2B_Rs in lymphoid tissues of EAE mice (Lazarus et al., 2011). Pharmacological blockade of A_2B_R (via A_2B_R antagonists MRS-1754 and CVT-6883) relieved the symptoms and pathological changes of EAE, accompanied by a suppression of IL-6 production and Th17 cell differentiation. A promising work from Gonzalez-Fernandez et al. (2014) demonstrated that activation of A_3_R may contribute to oligodendroglial cell death in optic nerve and white matter ischemic damage. However, further investigations are required to fully understand the effects of A_2B_R and A_3_R in the myelination and demyelination processing.

## Adenosine Actions in Autism

The study of adenosine in regards to autism has been around for decades (Masino et al., 2013); however, only within the last 10 years have multiple lines of research revealed the important role of adenosine and ARs in ASD. Due to findings that adenosine action affects many various aspects of ASDs, such as neuronal activity, sleep and seizures, cognition, and anxiety, the adenosine signaling pathway may be primed to be a potential therapeutic target for ASD.

### Adenosine and Its Metabolism in ASD

A patient study from Masino et al. (2009) using a customized parent-based questionnaire indicated an observable relationship between activities that were predicted to increase adenosine levels in the brain and parental observations of relieved behavioral manifestations of ASD. Their results suggest, in general, that increased adenosine signaling activity can benefit sleep disorders, seizures, and social and behavioral dysfunction in ASD (Stubbs et al., 1982; Shen et al., 2008). Augmentation of adenosine signaling activity may serve as a novel therapeutic strategy for ASD with multiple potential beneficial effects in alleviating core symptoms and several comorbidities of ASD (Bottini et al., 2001). However, no direct evidence of dysregulated adenosine in the brain has been tested with respect to the manifestations of ASD (Hettinger et al., 2008). Adenosine metabolic enzymes were also found to have links to ASD. A reduced activity of ADA was reported in the sera of autistic children (Freitag et al., 2010), and a low-activity polymorphism (Asp8Asn) of ADA gene was found to be significantly associated with ASD patients (Ansari et al., 2017a; Amodeo et al., 2018). The above lines of research suggest that reduced ADA activity may be a risk factor for the development of ASD.

### Adenosine Receptors and ASD

The A_2A_Rs are found expressed predominantly in the caudate nucleus, where they exert their influence in ASD pathology (Ahmad et al., 2017a). Genetic studies reveal that one of the polymorphisms of *A_2A_R gene*, rs2236624-CC, has a nominal association with ASD and three others, rs3761422, rs5751876 and rs35320474, and influences phenotypic variability in ASD symptoms (Ahmad et al., 2017a). Interestingly, the A_2A_R gene is located on chromosome 22q11.23, and large 22q11.2 duplications and deletions were observed in individuals with ASD; this correlation suggests a possible role of A_2A_R variants in mediating phenotypic expression in ASD (Ahmad et al., 2017a).

Repetitive behaviors and restricted interests are commonly related to neurodevelopmental disorders which are diagnostic for ASD. As a means of exploring these phenotypes, BTBR T^+^ Itpr3tf/J (aka BTBR) mice can be utilized as an animal model of idiopathic autism due to their strong and consistent autism-relevant behaviors (Ahmad et al., 2017b; Ansari et al., 2017b). Studies showed that A_2A_R agonism *via* the adenosine analog CGS-21680 caused a reduction of repetitive and inflexible behaviors in these BTBR mice, whereas A_2A_R antagonist SCH-58261 treatment exacerbated repetitive behaviors. This antagonism was also seen to cause a decreased expression of IL-27 and IkB-α and an increased expression of NF-kB p65 and toll-like receptors, TLR2, TLR3, and TLR4 in the brain (Tanimura et al., 2010; Campbell et al., 2013; Lopez-Cruz et al., 2017). Correspondingly, other pharmacological studies have shown that activation of A_1_R and A_2A_R *via* co-administration of A_2A_R agonist CGS-21680 and A_1_R agonist CPA attenuates stereotypy (repetitive behaviors) in a second animal model of ASD in a dose-dependent manner (Zhu et al., 2011). A_2A_R inactivation also affects social behaviors and anxiety in the autism spectrum; A_2A_R knockout mice show an anxiety profile, higher levels of sociability, and a reduced sensitivity to social novelty (Matute et al., 2001). Pharmacological manipulation of A_2A_R can conversely potentiate motivation to work. Together, these studies support the notion that augmentation of AR activities in the CNS could be a promising therapeutic strategy for ASD.

In addition, a third player, A_3_R, was shown to have two rare coding variants (Leu90Val and Val171Ile) in a genetics study using four single-nucleotide polymorphisms (SNPs) representing common haplotypes of 958 families with autism. It was revealed that these variations were found at an increased rate in ASD cases (Alberdi et al., 2006). Further *in vitro* analysis revealed that these same coding variants, Val90-A_3_R and Ile171-A_3_R, link to an elevated 5-HT re-uptake activity which responds differently to agonism of the 5-HT transporter. This suggests that these hyperfunctional coding variants of A_3_R may have an impact on ASD risk (Alberdi et al., 2006; Chez et al., 2007).

## Adenosine and Related Neurotransmitters in ASD

Adenosine-related neurotransmitters, such as glutamate, dopamine, histamine, and others have all taken part in the initiation and progression of ASD. Under pathological conditions, glutamate can also damage white matter *via* an over-activation of NMDAR, which is seen in ischemia, anoxia, infection, and MS (Owley et al., 2006). Likewise, glutamate can also cause the damage and death of OLs via activating AMPAR or kainate receptors, which in turn detrimentally increases Ca^++^ permeability (Karadottir and Attwell, 2007). In addition, glutamate can also activate kainate receptor-linked microglia/macrophage action to cause OL damage (Hellings et al., 2017). However, inhibition of AMPAR or kainate receptors alone does not exert an effect on OLs or myelination. Together, these data suggest that glutamate facilitates migration but inhibits the proliferation of OPCs, which is similar to adenosine-mediated effects on oligodendroglial lineage cells. This suggests that the effect of glutamate in disease may be caused by adenosine. In some ASD individuals, glutamate levels were found to be abnormally high (Paval, 2017). Glutamate antagonists, such as amantadine and memantine, have been shown to be effective in ASD treatment, by improving memory, hyperactivity, irritability, language, social behavior, and self-stimulatory behavior (Paval, 2017; Lee et al., 2018).

**FIGURE 2:**
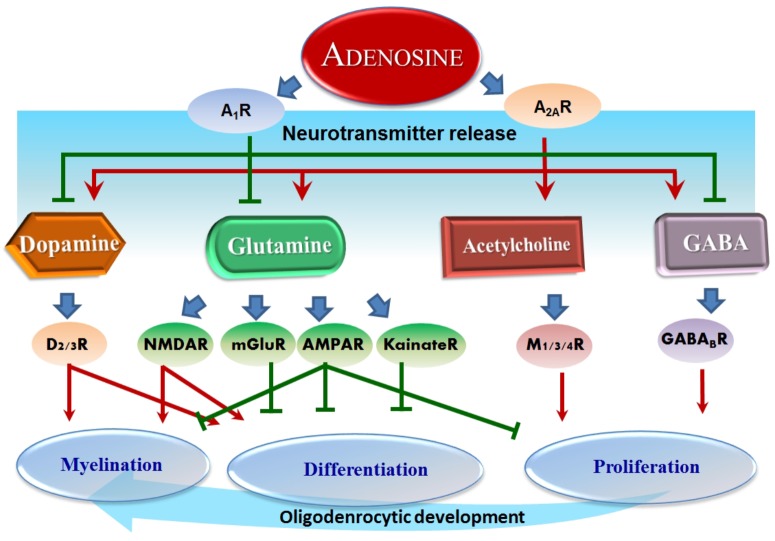
Adenosine-modulated neurotransmitters and their effects on OLs. Overview of adenosine effect on neurotransmitters: adenosine inhibits dopamine, glutamine, and GABA through A_1_R, whereas A_2A_R serves to conversely promote dopamine, glutamine, acetylcholine, and GABA. These neurotransmitters successively regulate further downstream receptors and ultimately several essential processes in oligodendrocytic development, such as proliferation, differentiation, and myelination. Red arrows represent a stimulation effect; Green bars represent an inhibition effect; Blue arrows show the directionality of effects.

Dopamine has a fundamental role in the brain and its role in ASD has already been thoroughly studied (Seeman, 2010; Mei et al., 2016a). Moreover, it has been shown that using antagonists for dopamine D_1_ receptors in mice revealed significant deficits in sociability and repetitive behaviors related to ASD. Small interfering RNA (siRNA)-mediated inhibition of dopamine D_2_ receptors in the dorsal striatum replicated ASD-like phenotypes (Mei et al., 2016b). Dopaminergic system deficits have been closely related to behavior skills such as analyzing, planning and prioritizing (Matson et al., 2011). Consistent with this, children with ASD revealed deficits in working memory and flexibility. Dopamine has a close relationship with social behavior, attentional skills, and perception, which are all related to ASD (Mei et al., 2016a). Recently, it was found that muscarinic M1 receptors negatively regulate OPC differentiation and their ablation can accelerate remyelination (Mei et al., 2016a). Notably, oligodendroglial development and myelination are complex programs under the control of multiple neurotransmitters. For instance, it was found that quetiapine, an antagonist for multiple neurotransmitters including dopamine, muscarine, and opioids, promoted OL differentiation and remyelination (Mei et al., 2016b), demonstrating that quetiapine may be a promising drug to be tested in future clinical trials. These facts reveal the importance of neurotransmitters in ASD and that targeting dopamine and/or glutamate signaling pathway effects on myelination is an effective strategy for ASD treatment. Currently, however, only two atypical antipsychotic drugs, risperidone and aripiprazole, are FDA-approved as clinically effective in improving ASD behavioral symptoms (Matson et al., 2011). Therefore, it may be a promising strategy to treat ASD by regulating adenosine, which will in turn affect the related neurotransmitters (Figure 2).

## Concluding Remarks

Increased evidence from clinical and basic research suggests a relationship between the adenosine signaling system and ASD. The broad actions of the adenosinergic system on neuroprotection, neuroinflammation, modulation of neurotransmissions, and regulation of glial function are involved in the core behavioral symptoms in ASD, irrespective of cognitive function. Therefore, adenosine-based strategies, through manipulating adenosine signaling pathways and affecting oligodendrocyte activities, may be utilized to therapeutically target core symptoms and comorbidities of ASD. Although adenosine’s effect is complex and pleiotropic, its effect on myelination can work through multiple neurotransmitter-induced pathways and improve behavior deficits in ASD, suggesting that this strategy is effective. However, focused studies on adenosine, oligodendroglia, and myelination are urgently needed to fully uncover the role of the adenosine system in ASD. While multiple adenosine receptor agonists are in clinical trials, therapeutic augmentation of adenosinergic signaling is ready to be tested for diverse potential benefits against multiple comorbidities on the autism spectrum.

## Author Contributions

H-YS and NH wrote the manuscript. JR and LX revised the manuscript. H-YS and LX designed the manuscript.

## Conflict of Interest Statement

The authors declare that the research was conducted in the absence of any commercial or financial relationships that could be construed as a potential conflict of interest.

## Abbreviations

5-HT: 5-hydroxytryptamine
A_1_R: adenosine A_1_ receptor
A_2A_R: adenosine A_2A_ receptor
A_2B_R: adenosine A_2B_ receptor
A_3_R: adenosine A_3_ receptor
ADA: adenosine deaminase
ADK: adenosine kinase
AMPAR: α-amino-3-hydroxy-5-methyl-4-isoxazolepropionic acid receptor
ASD: autism spectrum disorder
BBB: blood–brain barrier
cAMP: cyclic adenosine monophosphate
CB1R: cannabinoid receptor (type 1)
D_1_R: dopamine D1 receptor
D_2_R: dopamine D2 receptor
D_3_R: dopamine D3 receptor
EAE: experimental allergic encephalomyelitis
GABA: gamma-aminobutyric acid
IL: interleukin
MS: multiple sclerosis
NMDAR: *N*-methyl-D-aspartate receptor
NTs: nucleotide transporters
OL: oligodendrocyte
OPC: oligodendrocyte precursor cell
SAH: *S*-adenosyl-L-homocysteine
TLR: toll-like receptor
